# Isolation and Characterization of the New Mosaic Filamentous Phage VFJ Φ of *Vibrio cholerae*

**DOI:** 10.1371/journal.pone.0070934

**Published:** 2013-08-01

**Authors:** Qiuya Wang, Biao Kan, Ruibai Wang

**Affiliations:** State Key Laboratory for Infectious Disease Prevention and Control, Collaborative Innovation Center for Diagnosis and Treatment of Infectious Diseases, National Institute for Communicable Disease Control and Prevention, Chinese Center for Disease Control and Prevention, Beijing, P. R. China; University of Illinois at Urbana-Champaign, United States of America

## Abstract

Filamentous phages have distinguished roles in conferring many pathogenicity and survival related features to Gram-negative bacteria including the medically important *Vibrio cholerae*, which carries factors such as cholera toxin on phages. A novel filamentous phage, designated VFJΦ, was isolated in this study from an ampicillin and kanamycin-resistant O139 serogroup *V. cholerae* strain ICDC-4470. The genome of VFJΦ is 8555 nucleotides long, including 12 predicted open reading frames (ORFs), which are organized in a modular structure. VFJΦ was found to be a mosaic of two groups of *V. cholerae* phages. A large part of the genome is highly similar to that of the fs2 phage, and the remaining 700 bp is homologous to VEJ and VCYΦ. This 700 bp region gave VFJΦ several characteristics that are not found in fs2 and other filamentous phages. In its native host ICDC-4470 and newly-infected strain N16961, VFJΦ was found to exist as a plasmid but did not integrate into the host chromosome. It showed a relatively wide host range but did not infect the classical biotype O1 *V. cholerae* strains. After infection, the host strains exhibited obvious inhibition of both growth and flagellum formation and had acquired a low level of ampicillin resistance and a high level of kanamycin resistance. The antibiotic resistances were not directly conferred to the hosts by phage-encoded genes and were not related to penicillinase. The discovery of VFJΦ updates our understanding of filamentous phages as well as the evolution and classification of *V. cholerae* filamentous phage, and the study provides new information on the interaction between phages and their host bacteria.

## Introduction

Inovirus, which members are commonly called filamentous bacteriophages, is a special phage genus. Unlike the closely related archetypal bacteriophages which have a typical head-tail morphology and linear double-stranded DNA and releases their progeny by lysing the host cell, filamentous bacteriophages are long, thin filaments which contain a circular single-stranded DNA genome and reproduce without killing the host and even take part in the host’s life cycle [1], [2]. Filamentous bacteriophages are widespread among Gram-negative bacteria and have two major types of life cycles, exclusive episomal replication and chromosomal integration combined with episomal replication, the latter of which has been observed for temperate phages [1].

*Vibrio cholerae* is the causative agent of cholera, a deadly diarrheal disease. Several filamentous phages have been previously identified in *V. cholerae* [3]–[8]. Based on the gene sequences of the replication protein, pIII-like receptor-binding coat proteins, and the Zot-like protein, these phages can be clustered into three distinct branches represented by the CTX phage, fs2 phage, and a group of five other phages, KSF-1, VGJ, VSK, VSKK, and fs1 [6]. Filamentous phages have provided many key phenotypes to their *V. cholerae* hosts, including virulence attributes. The most well-studied *V. cholerae* phage, CTXΦ, exists as a prophage in toxigenic *V. cholerae* and encodes the cholera toxin (CT); thus, it plays a crucial role in the pathogenicity of *V. cholerae* and its transfer between cells [9]–[13]. Phage 493 is conjectured to have played some role in the emergence of the O139 epidemic serotype of *V. cholerae* [5]. Phage fs2 can horizontally transfer its *rstC* gene to the classical strain and influence the assembly of fimbriae in an animal model [14]. Almost all of these phages have been proven to be integrated into the host chromosome [6], [9], [15], [16].

In this study, we isolated a novel plasmid, designated VFJΦ, from the *V. cholerae* O139 serogroup strain ICDC-4470 (Amp^r^, Kan^r^). The sequence of this plasmid was highly similar to that of phage fs2, except for a fragment of approximately 700 bp between the att site and the first open reading frame (ORF). Unlike the other *V. cholerae* phages mentioned above, VFJΦ is an episomally replicating phage and has distinct biological effects on its host. Thus, we present here the whole nucleotide sequence and genome structure of this new phage, as well as its host range and biological functions.

## Materials and Methods

### Strains, Plasmids, and Media

The bacterial strains and plasmids used in this study are presented in Table 1. All strains were grown in Luria-Bertani (LB) medium. Antibiotics were added, when necessary, at the following concentrations: ampicillin, 100 µg/ml, and kanamycin, 50 µg/ml. The presence of genes encoding CT, toxin-coregulated pili (TCP) and mannose-sensitive hemagglutinin (MSHA) pilus was ascertained using PCR assays (primers listed in Table 1).

**Table 1:** Bacterial strains, their susceptibilities to VFJΦ and primers used in this study.

Strains and primers	Description	Year of isolation	Location (province)	*ctxAB*	*tcpA*	*mshA*	Susceptibility to VFJΦ
*V. cholerae* strains							
ICDC-4470	O139	1997	Fujian	+	+	+	/
VC4360	non-O1/non-O139	2010	Xinjiang	–	–	+	2.2×10^−6^
VC4361	non-O1/non-O139	2010	Xinjiang	–	–	+	5×10^−7^
VC4362	non-O1/non-O139	2008	Sichuan	+	+	+	6.6×10^−7^
O395	O1, classical	1964	India	+	+	+	ND^a^
569B	O1, classical	1948	India	+	+	+	ND
1119	O1, classical	1964	Dacca	+	+	+	ND
16017	O1, classical		India	+	+	+	ND
N16961	O1, El Tor, Inaba	1971	Bangladesh	+	+	+	1.71×10^−6^
VC2507	O1, El Tor, Inaba	1995	Shandong	–	+	+	1.5×10^−5^
VC2065	O1, El Tor, Inaba	1984	Shannxi	+	+	+	ND
VC1820	O1, El Tor, Inaba	2001	Xinjiang	+	+	+	3.3×10^−7^
VC3246	O1, El Tor, Ogawa	1977	Shandong	–	+	+	4.3×10^−6^
VC4421	O1, El Tor, Ogawa	1993	Liaoning	–	+	+	4×10^−6^
40–42	O1, El Tor, Ogawa	1977	Zhejiang	–	+	+	ND
VC126	O1, El Tor, Ogawa	2004	Liaoning	–	–	–	ND
VC1718	O1, El Tor, Ogawa	1999	Chongqing	+	+	+	1.5×10^−7^
VC1707	O1, El Tor, Ogawa	2004	Liaoning	+	+	+	1.3×10^−5^
VC4395	O139	1994	Zhejiang	+	+	+	6.7×10^−6^
VC3772	O139	1993	Xinjiang	+	+	+	6.7×10^−7^
VC4074	O139	1994	Xinjiang	–	–	+	2.5×10^−6^
VC4425	O139	1994	Beijing	–	–	+	ND
Other strains							
94-9-3	*V. mimicus*	1994	Jiangsu	–	–	+	2.5×10^−6^
38257	*V. mimicus*			–	–	+	2.3×10^−6^
SX-4	*V. vulnificus*	2009	Shanxi	+	–	+	1×10^−6^
CMCP6	*V. vulnificus*			–	–	+	4.1×10^−6^
VP803	*V. parahaemolyticus*	2012	Zhejiang	–	–	+	ND
VP355	*V. parahaemolyticus*	2011	Shanghai	–	–	+	ND
Shanghai18-96	*V. metschnikovii*	1996	Shanghai	–	–	+	1.8×10^−6^
Shanghai8-90	*V. metschnikovii*	1990	Shanghai	–	–	+	ND
93-9-3	*V. fluvialis*	1993	Jiangsu	–	–	+	ND
Shanghai13090	*V. furnissii*	1990	Shanghai	–	–	+	ND
Primers							
VFJ-U	TAAAGCAGCGTTAATCGTTGCCTC						This study
VFJ-L	CACATCCAACCAACAACGGTAGAG						This study
CT-F-RT	CTCTTCCCTCCAAGCTCTATGC						This study
CT-R-RT	TGGGGTGCTTGATGAACAATTAC						This study
ctxB-L	GATGAAGGATACCCTGAGGATTGC						This study
rtxA-U	ATGGCGTGGTCAGACGTGGTTCAC						This study
vc1465-L	TGTCATGATGGTCGGTTATGATGC						This study
vc1461-U	AGAAAACCCCGAGTGAAAGCGTGC						This study
CT-U	CTCAGACGGGATTTGTTAGGCACG						This study
CT-L	TCTATCTCTGTAGCCCCTATTACG						This study
TCP-U	AAAACCGGTCAAGAGGG						This study
TCP-L	CAAAAGCTACTGTGAATGG						This study
msha400F	AAGATGAAATCGGGTTG						This study
msha400R	TATCTGGCGACGCTTGC						This study
msha-1	AGCGAAAGCGAATAGTGG						This study
msha-4	GTGGTTACCACCGCAAAGG						This study

a ND, no Kan^r^ colonies detected.

### Isolation of Phage VFJ

ICDC-4470 was grown in 200 ml of LB broth in a 500 ml flask with shaking (240 rpm) at 37°C overnight. After centrifugation, the supernatants were filtered through 0.22-µm pore size filters (Millipore). Approximately 20 µl of the filtered supernatant was plated onto solid LB to check for sterility, and the remainder was used to precipitate the phage particles by the addition of NaCl and polyethylene glycol to final concentrations of 3 and 5% (wt/vol), respectively. The mixture was incubated on ice for 2 hours and centrifuged at 12,000×*g* for 20 min. The phage-containing pellet was resuspended in 1 ml of SM buffer (100 mM NaCl, 8 mM http://cshprotocols.cshlp.org/lookup/doi/10.1101/pdb.caut510MgSO4, 50 mM Tris-Cl, 0.002% gelatin).

### DNA Isolation and Manipulation

*V. cholerae* total DNA and genomic DNA of phage particles were prepared using a phenol-chloroform extraction method as described by Sambrook *et al*. [17]. Plasmid DNA was prepared using a QIAprep Spin Miniprep Kit (Qiagen, Hilden, Germany). DNA restriction and modification enzymes were used according to the manufacturer’s instructions (Takara, Dalian, China). Southern blot analysis was performed using random primer DIG DNA Labeling and Detection Kit (Roche Molecular Biochemicals, Mannheim, Germany) and the whole VFJ plasmid labeled as the specific digoxigenin probe. The entire sequence of the VFJΦ genome was assembled by combining data obtained from the sequencing of cloned *Hinc*II enzyme digested fragments of the VFJ plasmid in the pUC19 vector and from subsequent direct sequencing of the purified genomic DNA of the phage by primer walking.

### Electron Microscopy

Purified phage particles and *V. cholerae* cells were negatively stained with 2% (wt/vol) uranyl acetate and mounted on freshly prepared Formvar grids, which were examined with a Tecnai G2 Spirit transmission electron microscope (FEI, Hillsboro, OR, USA.).

### Infection Assay

The receptor strains were inoculated into 5 ml LB broth, and incubated statically at 30°C for 24 h. Subsequently, aliquots (400 µl) of pure phage VFJΦ were mixed with 100 µl cultures of the receptor strain. The mixture was incubated for 3 h in 5 ml fresh LB broth to allow for infection. After dilution, the infected cultures were plated onto solid LB plates with or without kanamycin, and the proportions of total cells infected were calculated (number of colonies growing on kanamycin plates divided by the total number of colonies recovered). All of the N16961 colonies and some of the other infected strains growing on kanamycin plates were also ascertained using PCR assays with VFJΦ primers.

### Quantitative Real-time PCR (qRT-PCR)

DNA isolated from the phage particles were 10-fold serially diluted and used as the template. The primer pairs VFJ-U with VFJ-L and CT-F-RT with CT-R-RT were used for the amplification and comparative quantitation of the copy numbers of VFJΦ and CTXΦ. The reactions were performed using the SYBR premix Ex Taq™ (TaKaRa).

### Motility Test

Wild-type and infected strains were incubated in 3 ml of LB broth at 37°C overnight. Thereafter, 100 µl of culture was transferred into 5 ml of fresh LB broth and incubated until an OD_600_ of 0.6 was reached. Approximately 10 µl of culture was directly pierced into 0.3% LB swim agar plates using a pipette tip and cultured at 37°C for 24 h, and then the diameters of each colony was measured.

### Penicillinase Production Test

The procedure described by Gots and modified by Haight and Finland [18] was used to test for penicillinase production of the strains infected by VFJΦ in this study. Briefly, the sensitive *Escherichia coli* strain DH5α was inoculated on the entire Mueller-Hinton agar surface, and the susceptibility testing discs of ampicillin (10 µg) were placed onto the centre. The test VFJΦ-infected strains and positive control strain DH5α-pUC18 (Amp^R^) were streaked from the susceptibility testing disc to the edge of the plate. After 16 to 18 h of incubation, the resulting zones of inhibition were observed.

### Nucleotide Sequence Accession Number

The sequence of the VFJΦ genome has been deposited in GenBank (accession number KC357596). The ORFs were predicted by using the GeneMark software [19].

## Results

### Nucleotide Sequence and Genomic Organization

**Figure 1:**
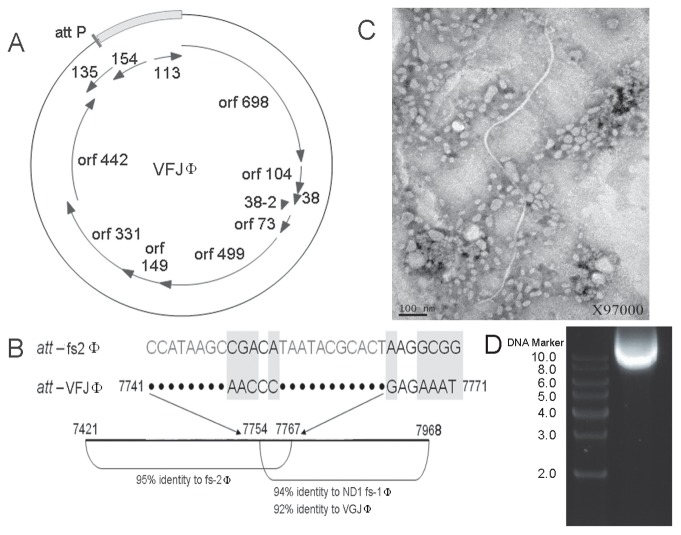
Circular diagram of the VFJ genome and predicted ORFs (A). **The non-identical region is marked with a grey box.** Sequence alignment of the *attP* regions of VFJΦ and fs2 Φ (accession no. NC_001956) and schematic representation of adjacent areas (B). Electron microscopy image of VFJΦ precipitated from the ICDC-4470 culture supernatant (C).

The genomic DNA, designated VFJ and obtained as a plasmid from strain ICDC-4470, migrated to a position corresponding to a size of approximately 8****kb on an agarose gel (Fig. 1D) and could be digested into 2 kb and 6 kb fragments by the restriction enzyme *Hinc*II, indicating that the DNA was double stranded. The complete genome was 8555 nucleotides long, with a G+C content of 44.23 mol %, which deviates from that of the host *V. cholerae* (47.49 mol %). The ATG site of orf 698 was taken as the zero coordinate of the sequence. The sequence between nt 1 and 7765, corresponding to 90.8% of the whole genome, was 97% identical to the genomic sequence of the *V. cholerae* phage fs2. The sequence from nt 7301 to 8179 was 81% similar to that of vibrio phages ND1 fs-1, VCY, and VGJ, whereas the last 375 nucleotides exhibited no homology with other known sequences (Fig. 1A).

Overall, 12 ORFs were predicted in the VFJ genome by ORF prediction and BLAST searching (Fig. 1A, Table 2), among which 9 ORFs were located in the region observed to be highly homologous to the fs2 phage. Three phage structural proteins of VFJΦ, pV, pIX, and pVIII, encoded by orf 104, orf 38-2, and orf 73, respectively, were 100% similar to those of fs2. However, the homologies of pVII and pIV were both lower than 90%. Four residues at the C terminus of pVII, encoded by orf 38, were completely different from the equivalent residues in fs2. The pIV protein, corresponding to orf 442, was 58 amino acids shorter than that of fs2 due to the deletion of a single T nucleotide at 7301 and the presence of a stop codon at 7302. Usually, the structure of a virion filament tube consists of thousands of copies of pVIII in a helical arrangement. Two pairs of proteins, pVII-pIX and pIII-pVI, make up the two ends of the filament. The pVII-pIX proteins are incorporated into the virion at the initiation step of assembly and are the first to be extruded from the cell. The pIII and pVI proteins are required for the structural stability of the virion and for the termination of assembly. PIII also mediates the entry of the phage into the host cell. In addition, proteins pI, pIV and pXI form a transport complex spanning the inner and outer membranes. Thus, the differences in the structural proteins between VFJΦ and fs2 were observed primarily in the head cap and transporter.

**Table 2:** Putative ORFs in the VFJ phage genome and their homologs.

VFJ	Position of transcription	Nucleotides(bp)	Description*	Homologous protein	Matchingbases (bp)	Similarity of proteins (%)
orf 698	1–2097	2097	Replication protein	orf 716 of fs2	2057	94
orf 104	2150–2464	315	Gene V (ssDNA-binding protein)	orf 104 of fs2	305	100
orf 38	2475–2591	117	Gene VII (minor coat protein)	orf 39 of fs2	111	82
orf 38–2	2588–2704	117	Gene IX protein (minor coat protein)	orf 38 of fs2	116	100
orf 73	2729–2950	222	Gene VIII protein (major coat protein)	orf 73 of fs2	222	100
orf 499	3028–4527	1500	Receptor binding	orf 498 of fs2	1488	97
orf 124	4534–4905	372	Gene VI protein	orf 116 of fs2	349	91
orf 361	4910–5995	1086	Gene I protein (phage morphogenesis)	orf 361 of fs2	1050	97
orf 442	5976–7304	1329	Gene IV protein (phage morphogenesis)	orf 500 of fs2	1312	86
orf 135	7719–7312	408	Putative transcription regulator	putative transcription regulator protein of VEJ phi	325	89
orf 154	8107–7643	465	Regulatory proteins	hypothetical protein of VCY-phi	384	79
orf 113	8215–1	342	Translation regulation	hypothetical protein VV1_0070 of *V. vulnificus* CMCP6	148	57

* Putative function based on that of the homologous protein.

In the region that was non-homologous to fs2, three ORFs were identified. Orf 135 and orf 154 were found to be homologous to the regulatory proteins of two other *V. cholerae* filamentous phages, VEJ and VCYΦ, respectively. The last ORF, orf 113, was found to be homologous to a hypothetical protein of *Vibrio vulnificus*, CMCP6, which contains an ASCH domain [20] and may be associated with translational regulation. The promoter region of the first ORF, orf 698, also lies in this complete nonhomologous region, although the proteins produced by orf 698 of VFJ and fs2 have up to 94% similarity.

### Purification and Determination of Copy Number of VFJΦ Relative to CTXΦ

We precipitated phage particles from cell-free culture supernatants of ICDC-4470 without any inducer and could detect filamentous phage structures by electron microscopy that were approximately 1.4 µm in length and 7 nm in width (Fig. 1). After full digestion with DNAse and RNAse, the nucleic acids were extracted from the purified phage particles and amplified with primers designed based on the VFJ plasmid. The extracted nucleic acid could not be cut by *Hinc*II but was sensitive to treatment with S1 nucleases, indicating that the nucleic acid was single-stranded DNA. All of the sequences of the PCR products amplified from the phage nucleic acid were the same as those amplified from the plasmid. Moreover, precipitates from the cell-free culture supernatants of ICDC-4470 could infect other strains, including DH5α and N16961, and VFJΦ could be re-extracted from these recipients as a plasmid. Based on these results, we concluded that the VFJ plasmid is the replication form of the phage.

**Figure 2:**
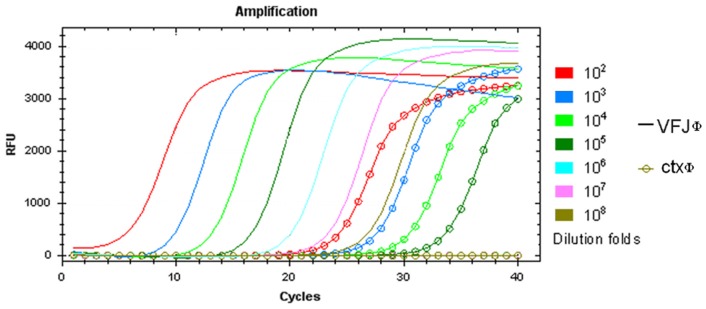
qRT-PCR graph showing relative copy numbers of VFJΦ and CTXΦ. DNA was extracted from the pellets of cell-free cultures of ICDC-4470, 10-fold serially diluted, and amplified using VFJ- and CTX- specific primers.G.

ICDC-4470 was positive for the *ctx* gene; therefore, it could also release CTXΦ. By using qRT-PCR, the relative copy numbers of VFJΦ and CTXΦ released by ICDC-4470 in the culture supernatant without any inducer were compared (Fig. 2). Large differences were observed in the copy numbers of the two phages, with the copy number of VFJΦ being approximately 10^5^ times that of CTXΦ.

### Integration of VFJΦ

**Figure 3:**
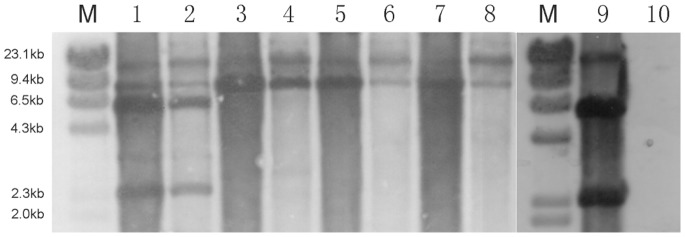
Southern hybridization analysis of the integration of the VFJ phage into host chromosomes. Genomic DNA of ICDC-4470 and control VFJ plasmid DNA were digested with *BamH*I, *EcoR*I (no cut site in VFJ), *Nde*I (only one cut site), and *Hind*III (two cut sites). Lanes 1, 3, 5 and 7 correspond to ICDC-4470 DNA digested with *Hind*III, *Nde*I, *EcoR*I, and *BamH*I, while lanes 2, 4, 6 and 8 correspond to plasmid DNA digested with the same respective enzymes. The genomic DNA of N16961-VFJ and wild-type N16961 not infected by VFJ were also digested by *Hind*III and hybridized (lanes 9 and 10, respectively).

The VFJ genome contains a 20 bp *att*-like sequence (*attP*) (Fig. 1B), with only four base pairs that differ with respect to fs2 in the central region. The *attP* sequence is located at the junction of the fragments that are identical and non-identical to the fs2 phage. To determine if VFJΦ integrates into the host chromosome, the genomic DNA of ICDC-4470 and that of NI6961-VFJ were extracted and blotted with a digoxigenin-labeled VFJ probe. The results indicated that the patterns for ICDC-4470, N16961-VFJ, and the plasmid control were exactly the same (Fig. 3). The theoretical pattern that should appear if VFJΦ had integrated into the host chromosome was not observed. Additionally, the RS regions flanked by the CTX core element (VC1456-VC1461, coding *ctxAB*, *ace*, *zot* and *cep* genes) are hot integration sites for *V. cholerae* phages, such as fs2. Using the primer pairs ctxB-Lwith rtxA-U and vc1465-L with vc1461-U, the two flanking regions were amplified and sequenced. However, there were still no signs that VFJΦ had integrated. Therefore, it can be concluded that under normal culture conditions, in its native host strain ICDC-4470 and newly infected strain N16961-VFJ, VFJΦ exists only in the form of a plasmid without chromosomal integration.

### Host Range

The VFJ phage could infect all of the tested non-O1/non-O139 *V. cholerae* strains (3 of 3), *V. mimicus* strains (2 of 2), and *V. vulnificus* strains (2 of 2) (Table 1). Most of the O1 El Tor biotype (7 of 10) and O139 serogroup *V. cholerae* strains (3 of 4) and the *V. metschnikovii* strains (1 of 2) were also sensitive, and the susceptibility did not differ substantially among them. In contrast, the four O1 classical biotype *V. cholerae* strains, two *V. parahaemolyticus* strains and two *V. fluvialis* strains that were tested could not be infected by VFJΦ. The susceptibility of *V. cholerae* strains to VFJΦ were highly similar to that of the fs2 phage, for which above 70% of the El tor biotype and O139 serogroup strains could be infected by fs2Φ, whereas the proportion of the classical biotype strains that could be infected were lower than 8% [21].

### Impact of VFJΦ Infection on Host Cell

**Figure 4:**
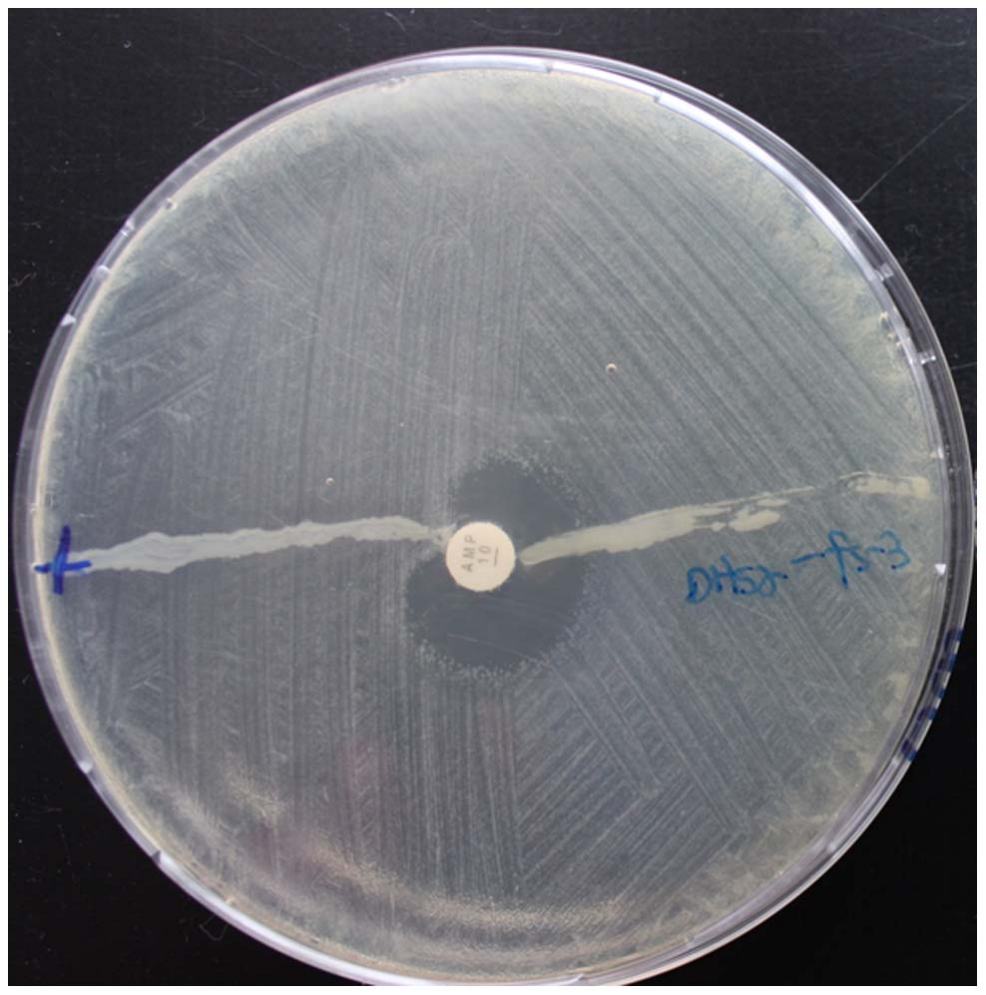
Microbiological assay of penicillinase production of the VFJΦ-infected *E. coli* strain DH5α. +indicates the positive control strain DH5α with pUC18 plasmid which carries the *amp* gene and can produce penicillinase.

Strain ICDC-4470, the native host of VFJΦ, is ampicillin- and kanamycin- resistant. After infection of VFJΦ, the recipient strains, including *E. coli* DH5α, *V. cholerae* strains, and all of the other tested *Vibrio* strains, also obtained low-level ampicillin resistance and high-level kanamycin resistance. The infected strains could tolerate 50 mg/ml ampicillin and 100 mg/ml kanamycin but could not grow in medium containing 100 mg/ml ampicillin. All of the kanamycin-resistant colonies were VFJ positive in PCR assays. The host spectrum of resistance to other antibiotics did not change by the infection with VFJΦ. In the microbiological assay of penicillinase, streaking of VFJΦ-infected DH5α on the plate did not affect the zone of inhibition of the indicator strain DH5α; meanwhile, the positive control strain DH5α-pUC18, which carries the Amp gene, made the zone of inhibition almost completely disappear (Fig. 4). This result clearly revealed that the ampicillin resistance caused by VFJΦ infection was not related to penicillinase.

**Figure 5:**
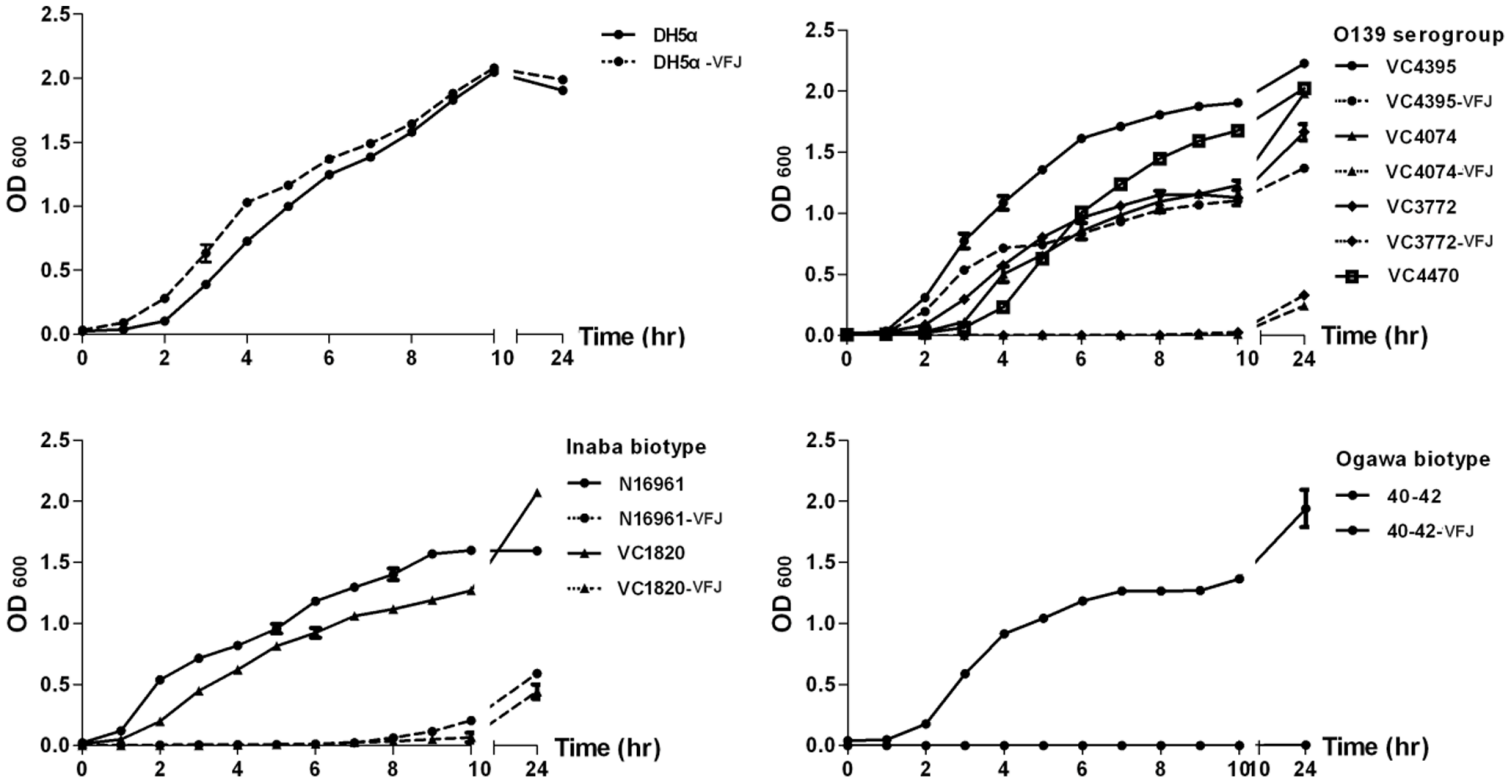
Growth curves of *E. coli* strain DH5α and three types of *V. cholerae* strains before and after infection by the VFJ phage.

**Figure 6:**
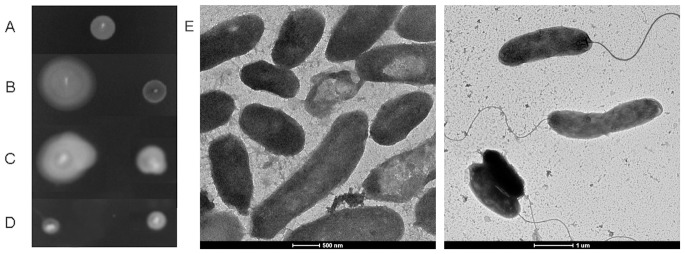
Motility experiments on swim plates (0.3% soft agar) and electron microscopy images showing the change in the flagella between the wild-type strain and the VFJ-infected strain. A. Strain ICDC-4470; B. VC4395 and VC4395-VFJ; C. N16961 and N16961-VFJ; and D. DH5α and DH5α-VFJ. E. Electron microscopy images of N16961 (right) and N16961-VFJ (left).

The second impact of VFJΦ infection on host cells was obvious growth inhibition, which was observed for two biotypes of the O1 serogroup and the O139 serogroup strains (Fig. 5). The colonies of these infected strains on LB agar cultured overnight were very small, and did not obviously grow when cultured in liquid LB broth during the initial ten hours, except for strain VC-4395-VFJ, which grew more rapidly than the other strains. However, the growth rate of the natural VFJ host strain ICDC-4470 was comparable to that of the other wild-type *V. cholerae* strains, and growth inhibition was not observed for the *E. coli* strain DH5α. The purified VFJ phage formed no turbid plaques when spotted on sensitive strains, and thus the inhibition was not the consequence of phage release and cell lysis. Additionally, on soft swim agar, ICDC-4470, VFJΦ-infected N16961 and 4395 strains could not form diffuser rings, indicating that these bacteria had lost mobility (Fig. 6A–D). Electron microscopy also revealed that the wild-type N16961 strain had a very long sheath-covered monotrichous flagellum, whereas the VFJ-infected strain lost this structure (Fig. 6E).

## Discussion

A number of *V. cholerae* filamentous phages have been reported. Because of their mosaic structures, phylogenetic analyses to assess the evolutionary relatedness and divergence among the phages have been performed at the level of individual genes, rather than the whole genomes [6], [22]. Based on the sequences of three essential genes encoding the replication protein, Zot-like protein and pIII-like protein that are distributed in three genomic regions, the *V. cholerae* phages can be divided into three branches: CTXΦ, fs2Φ, and a group of five other phages [6]. Understandably, CTXΦ is a distinct branch which uses TCP as receptor. However, phage fs2 also has diverged from other phages in all three phylogenetic trees even though it uses the same receptor MSHA pili as KSF-1, fs1, VSK, VSKK, and VGJ phages. Based on this phylogenetic method, VFJΦ should be grouped with fs2 since the similarities of all three VFJΦ genes to those of fs2 were above 94%. It is worth noting that downstream of the *att* site of the VFJ genome, the sequences are highly similar to those of VEJ and VCYΦ, which were isolated recently and found to be highly similar to VGJ group phages [7], [23]. Therefore, VFJΦ is a mosaic of these two groups of *V. cholerae* phages. In addition, orf 342 was the only ORF that did not match any other phage proteins and had the lowest similarity at the protein level, with 57% homology to a hypothetical protein of *V. vulnificus*, suggesting that recombination may have occurred more than once in the VFJΦ genome.

Generally, the genomes of filamentous phages are organized in a modular structure in which functionally related genes are grouped. In the best-studied members of the *Inovirus* genus, at least three functional modules are generally characterized: replication, structural and assembly modules [9]. The putative ORFs of VFJΦ are also organized in this manner. The replication module is composed of orf 698. The putative structural and assembly modules consist of orf 104 to orf 422, which encode the capsid proteins of the filamentous phage and the proteins required for viral particle packaging and secretion. However, after the *attP* site, the sequence of VFJ is completely different from that of fs2. In this region, fs2 carries the *rstC* gene and a large non-coding sequence. The *rstC* gene is the most notable feature of the fs2 genome and confers important biological function to this phage, namely the capacities to horizontally transfer the *rstC* gene and to affect the assembly of fimbriae *in vivo* [14]. The large non-coding sequence contains stem-loop structures and is suspected to correspond to the intergenic (IG) region of coliphages [21]. In contrast to fs2, VFJ does not carry the *rstC* gene in this region. Instead, VFJ contains a three-gene regulatory module, and as is typical for regular genes, orfs 408 and 465 are transcribed in the opposite direction of the other genes. This structure does not match that of episomally replicating filamentous phages, which replicate unabated after infecting the host cell, and their genomes do not encode regulatory proteins. This regulatory module is common in chromosomally integrated filamentous phages, which tightly control their gene expression and replication and encode transcriptional regulators to inhibit the transcription of the replication protein and virion genes [1], [24]. Under the culture condition without inducer, we did not detect chromosomal integration of VFJΦ in its native host strain ICDC-4470 and newly infected strain N16961-VFJ. There are two possible explanations for this outcome, one of which is that the mutant *att*-like sequence prevents integration. Besides the four base pairs in the central region of the 20 bp *att* site, the complete downstream flanking region of the *att* site of VFJΦ differs with respect to fs2. Therefore, VFJΦ is precluded from integrating into the host chromosome precisely as tha same manner as fs2. The regulatory module of the VFJΦ genome is a relic of its chromosomally integrated ancestor phage. The other possible explanation is that VFJΦ can integrate into certain *V. cholera* genomes or even that it can integrate into ICDC-4470 under specific conditions at which time its replicatory module may play a role in strictly controlling replication and gene expression.

The infected hosts of filamentous phages typically remain viable, and phages are continuously assembled and extruded from the cell in a concerted process. In addition, phage infection does not usually have a serious effect on cell growth; neither the CTXΦ phage of *V. cholerae,* which produces less than one phage particle per cell per hour, nor Ff phages, which produce hundreds of particles per cell per hour, substantially affecting the growth of the host [9]. It is notable that the bacterial strains infected with VFJΦ exhibited substantial growth inhibition. In contrast to CTXΦ, VFJΦ produced a high number of progeny. It is possible that the replication and release of this large number of phages required most of the energy of the host bacterium, thereby inhibiting the replication and growth of the host. Nonetheless, the *E. coli* strain DH5α infected with this phage grew normally, and the natural host of VFJΦ, ICDC-4470, also did not exhibit growth inhibition. In addition, the growth of one of the other tested strains was only partially inhibited, suggesting that the growth inhibition mechanism is species-specific and that an unknown mechanism exists that can relieve this growth inhibition.

VFJΦ also suppressed flagellum formation in the host. The flagellum is necessary for mobility and is known to contribute to virulence [25], [26]. All of the VFJ-infected strains, including its natural host, had no flagella and could not swim. Compared with the growth inhibition, flagella inhibition did not seem to be strain-specific. The inhibition of growth and flagellum formation also did not always occur simultaneously in one infected strain, implicating that the involvement of different mechanisms.

Another novel finding of this study is that along with the phage infection, the recipient strain acquired antibiotic resistance. Specifically, the VFJ phage could mediate the horizontal transfer of antibiotic resistance. More interesting is the observation that the transferred resistance was limited to two antibiotics, ampicillin and kanamycin. All of the predicted ORFs of VFJΦ have defined homologies, and none of them has been reported to be related with antibiotic resistance. Therefore, the antibiotic resistances are unlikely to be directly provided by the phage genes. Microbiological assays confirmed that these resulting drug-resistant VFJΦ strains did not produce penicillinase. Bacteriophages infect by delivering their genetic material into cells and mature by packaging the genome in capsids for release. The procedures are mediated by a DNA channel called “portal protein” [27], [28], which is a the transport complex formed by the proteins pI, pIV and pXI of filamentous phage. During viral maturation, the protein complex displays extraordinary structural plasticity and undergoes dramatic conformational changes [28]. We hypothesize that the ampicillin and kanamycin resistance obtained by the VFJΦ infected strains may be relate to this type of channel or involve phage infection-induced changes to some of the membrane structures, such as drug resistance pumps or efflux systems.

In conclusion, we have identified and characterized a novel mosaic filamentous phage of *V. cholerae*, VFJΦ, with a wide host range and varied effects on host cells. The discovery of this phage has provided new insights into the classification and evolution of *V. cholerae* phages and their interaction with host bacteria.

## References

[1] Rakonjac J, Bennett NJ, Spagnuolo J, Gagic D, Russel M (2011) Filamentous bacteriophage: biology, phage display and nanotechnology applications. *Curr Issues Mol Biol* 13: 51–76. 21502666

[2] Rice SA, Tan CH, Mikkelsen PJ, Kung V, Woo J, et al (2009) The biofilm life cycle and virulence of *Pseudomonas aeruginosa* are dependent on a filamentous prophage. *Isme J* 3: 271–282. 19005496 10.1038/ismej.2008.109 PMC2648530

[3] Honma Y, Ikema M, Toma C, Ehara M, Iwanaga M (1997) Molecular analysis of a filamentous phage (fsl) of *Vibrio cholerae* O139. *Biochim Biophys Acta* 1362: 109–115. 9540841 10.1016/s0925-4439(97)00055-0

[4] Kar S, Ghosh RK, Ghosh AN, Ghosh A (1996) Integration of the DNA of a novel filamentous bacteriophage VSK from *Vibrio cholerae* 0139 into the host chromosomal DNA. *FEMS Microbiol Lett* 145: 17–22. 8931321 10.1111/j.1574-6968.1996.tb08550.x

[5] Jouravleva EA, McDonald GA, Garon CF, Boesman-Finkelstein M, Finkelstein RA (1998) Characterization and possible functions of a new filamentous bacteriophage from *Vibrio cholerae* O139. *Microbiology* 144 (Pt 2): 315–324. 10.1099/00221287-144-2-315 9493369

[6] Faruque SM, Bin Naser I, Fujihara K, Diraphat P, Chowdhury N, et al (2005) Genomic sequence and receptor for the *Vibrio cholerae* phage KSF-1phi: evolutionary divergence among filamentous vibriophages mediating lateral gene transfer. *J Bacteriol* 187: 4095–4103. 15937172 10.1128/JB.187.12.4095-4103.2005 PMC1151723

[7] Xue H, Xu Y, Boucher Y, Polz MF (2011) High frequency of a novel filamentous phage, VCY phi, within an environmental *Vibrio cholerae* population. *Appl Environ Microbiol* 78: 28–33. 22020507 10.1128/AEM.06297-11 PMC3255608

[8] Nakasone N, Honma Y, Toma C, Yamashiro T, Iwanaga M (1998) Filamentous phage fs1 of *Vibrio cholerae* O139. *Microbiol Immunol* 42: 237–239. 9570290 10.1111/j.1348-0421.1998.tb02277.x

[9] Campos J, Martinez E, Suzarte E, Rodriguez BL, Marrero K, et al (2003) VGJ phi, a novel filamentous phage of *Vibrio cholerae*, integrates into the same chromosomal site as CTX phi. *J Bacteriol* 185: 5685–5696. 13129939 10.1128/JB.185.19.5685-5696.2003 PMC193952

[10] McLeod SM, Kimsey HH, Davis BM, Waldor MK (2005) CTXphi and *Vibrio cholerae:* exploring a newly recognized type of phage-host cell relationship. *Mol Microbiol* 57: 347–356. 15978069 10.1111/j.1365-2958.2005.04676.x

[11] Lencer WI, Tsai B (2003) The intracellular voyage of cholera toxin: going retro. *Trends Biochem Sci* 28: 639–645. 14659695 10.1016/j.tibs.2003.10.002

[12] Das B, Bischerour J, Barre FX (2011) Molecular mechanism of acquisition of the cholera toxin genes. *Indian J Med Res* 133: 195–200. 21415494 PMC3089051

[13] Davis BM, Waldor MK (2003) Filamentous phages linked to virulence of *Vibrio cholerae* . *Curr Opin Microbiol* 6: 35–42. 12615217 10.1016/s1369-5274(02)00005-x

[14] Nguyen DT, Nguyen BM, Tran HH, Ngo TC, Le TH, et al (2008) Filamentous vibriophage fs2 encoding the *rstC* gene integrates into the same chromosomal region as the CTX phage [corrected]. *FEMS Microbiol Lett* 284: 225–230. 18503544 10.1111/j.1574-6968.2008.01200.x

[15] Campos J, Martinez E, Marrero K, Silva Y, Rodriguez BL, et al (2003) Novel type of specialized transduction for CTX phi or its satellite phage RS1 mediated by filamentous phage VGJ phi in *Vibrio cholerae* . *J Bacteriol* 185: 7231–7240. 14645284 10.1128/JB.185.24.7231-7240.2003 PMC296256

[16] Waldor MK, Mekalanos JJ (1996) Lysogenic conversion by a filamentous phage encoding cholera toxin. *Science* 272: 1910–1914. 8658163 10.1126/science.272.5270.1910

[17] Sambrook J, Fritsch EF, Maniatis T (2001) Molecular Cloning: A Laboratory Manual,(3nd ed.). Cold Spring Harbor Laboratory Press, Cold Spring Harbor, NY.

[18] Haight TH, Finland M (1952) Modified Gots test for penicillinase production. *Am J Clin Pathol* 22: 806–808. 14943698 10.1093/ajcp/22.8_ts.806

[19] Besemer J, Borodovsky M (1999) Heuristic approach to deriving models for gene finding. *Nucleic Acids Res* 27: 3911–3920. 10481031 10.1093/nar/27.19.3911 PMC148655

[20] Iyer LM, Burroughs AM, Aravind L (2006) The ASCH superfamily: novel domains with a fold related to the PUA domain and a potential role in RNA metabolism. *Bioinformatics* 22: 257–263. 16322048 10.1093/bioinformatics/bti767

[21] Ikema M, Honma Y (1998) A novel filamentous phage, fs-2, of *Vibrio cholerae* O139. *Microbiology* 144 (Pt 7): 1901–1906. 10.1099/00221287-144-7-1901 9695923

[22] Lawrence JG, Hatfull GF, Hendrix RW (2002) Imbroglios of viral taxonomy: genetic exchange and failings of phenetic approaches. *J Bacteriol* 184: 4891–4905. 12169615 10.1128/JB.184.17.4891-4905.2002 PMC135278

[23] Campos J, Martinez E, Izquierdo Y, Fando R (2010) VEJ{phi}, a novel filamentous phage of *Vibrio cholerae* able to transduce the cholera toxin genes. *Microbiology* 156: 108–115. 19833774 10.1099/mic.0.032235-0

[24] Waldor MK, Friedman DI (2005) Phage regulatory circuits and virulence gene expression. *Curr Opin Microbiol* 8: 459–465. 15979389 10.1016/j.mib.2005.06.001

[25] Tsou AM, Frey EM, Hsiao A, Liu Z, Zhu J (2008) Coordinated regulation of virulence by quorum sensing and motility pathways during the initial stages of *Vibrio cholerae* infection. *Commun Integr Biol* 1: 42–44. 19704787 10.4161/cib.1.1.6662 PMC2633796

[26] Bari W, Lee KM, Yoon SS (2012) Structural and functional importance of outer membrane proteins in *Vibrio cholerae* flagellum. *J Microbiol* 50: 631–637. 22923112 10.1007/s12275-012-2116-3

[27] Dube P, Tavares P, Lurz R, van Heel M (1993) The portal protein of bacteriophage SPP1: a DNA pump with 13-fold symmetry. *Embo J* 12: 1303–1309. 8467790 10.1002/j.1460-2075.1993.tb05775.x PMC413341

[28] Rishovd S, Marvik OJ, Jacobsen E, Lindqvist BH (1994) Bacteriophage P2 and P4 morphogenesis: identification and characterization of the portal protein. *Virology* 200: 744–751. 8178458 10.1006/viro.1994.1238

